# User-centred design, validation and clinical testing of an anti-choking mug for people with Parkinson’s disease

**DOI:** 10.1038/s41598-024-65071-8

**Published:** 2024-06-19

**Authors:** Roongroj Bhidayasiri, Araya Chaisongkram, Chanawat Anan, Warongporn Phuenpathom

**Affiliations:** 1 Chulalongkorn Centre of Excellence for Parkinson’s Disease and Related Disorders, Department of Medicine, Faculty of Medicine, Chulalongkorn University and King Chulalongkorn Memorial Hospital, Thai Red Cross Society, 1873 Rama 4 Road, Bangkok, 10330 Thailand; 2 The Academy of Science, The Royal Society of Thailand, Bangkok, 10300 Thailand

**Keywords:** Parkinson’s disease, Oropharyngeal dysphagia, Aspiration, Anti-choking mug, Chin-down manoeuvre, User-centred design, Diseases, Neurology

## Abstract

Oropharyngeal dysphagia, or difficulty initiating swallowing, is a frequent problem in people with Parkinson’s disease (PD) and can lead to aspiration pneumonia. The efficacy of pharmacological options is limited. Postural strategies, such as a chin-down manoeuvre when drinking, have had some degree of success but may be difficult for people who have other limitations such as dementia or neck rigidity, to reproduce consistently. Using a user-centred design approach and a multidisciplinary team, we developed and tested an anti-choking mug for people with PD that helps angle the head in the optimum position for drinking. The design reflected anthropometric and ergonomic aspects of user needs with features including regulation of water flow rate and sip volume, an inner slope, a thickened handle and a wide base, which promoted a chin-down posture when used. Prototype testing using digital technology to compare neck flexion angles (the primary outcome), plus clinical outcomes assessed using standard tools (Swallowing Clinical Assessment Score in Parkinson’s Disease (SCAS-PD) and Movement Disorder Society-Unified Parkinson’s Disease Rating Scale (MDS-UPDRS) Parts II and III), found significant improvements in a range of parameters related to efficient swallowing and safe drinking when using the anti-choking mug versus a sham mug.

## Introduction

Aspiration of thin liquids is a common problem in people with Parkinson’s disease (PD) who have oropharyngeal dysphagia, a condition characterised by difficulty initiating swallowing. The condition has an estimated pooled prevalence of 36.9% when assessed using subjective outcomes, but this increases to 57.3% when using objective instrumental examination, suggesting it is likely to be underestimated by people with PD themselves who may not perceive that they have swallowing difficulties when in fact objective tests confirm they do have some degree of oropharyngeal dysphagia^1,2^. Oropharyngeal dysphagia does not just affect people in the advanced stage of PD, when the prevalence is > 80%, it can in fact occur at any disease stage, even before the classic motor symptoms become apparent^3,4^. In an international, observational, cross-sectional study of over 400 people with PD, approximately one-third reported at least some degrees of chewing and swallowing problem with the overall score correlating well with other disability measures^5^. Most of the time, the aspiration of thin liquids is asymptomatic (micro-aspirations) or mild, consisting of frequent coughing, throat clearing, or gurgling with wet vocal quality; however, serious complications can occur, such as recurrent respiratory infections, with aspiration pneumonia being recognised as a leading cause of malnutrition, low immunity, or even death amongst people with advanced PD^6^. There are also wider implications as difficulty swallowing is a predictor of the need for a carer^7^. Effective management strategies that can be implemented easily in the person’s daily life are therefore needed.

Treatment of oropharyngeal dysphagia in PD remains a challenge for neurologists as there are often various contributing factors, including advancing age, coexisted disorders, and the presence of dementia, in addition to the long disease duration characteristic of PD, which may not respond to dopaminergic medications, particularly levodopa. A recent consensus on the treatment of dysphagia in PD highlights that a multidisciplinary approach is essential and should involve all allied health professionals, such as speech-language pathologists, dietitians, and clinical nutritionists, to ensure the individualised management of people with PD^8^.

A literature review revealed that pharmacological options for addressing this issue are limited and, in many cases, only partially effective, leaving a critical unmet need in the management of this condition. The reported high rates of aspiration of thin fluids and subsequent development of aspiration pneumonia, a common cause of death in individuals with PD, also provide further evidence that the current approach to dysphagia management is still inadequate, and is reported to be unsatisfactory by those affected by PD, carers, and healthcare professionals^6,9,10^. Swallowing therapy is recognised as a core element in the treatment of dysphagia in PD, comprising standard swallowing therapy, the use of liquid thickeners, posture strategies, skill-based therapy, Lee Silverman Voice Therapy, and electrical muscle stimulation. However, current evidence of swallowing therapy as a management strategy is low, mainly due to inconsistent definition and methodology, a lack of control group in many studies, with several excluding individuals with dementia.

Amongst the posture-focused strategies, the chin-down posture (also referred in some cases as the chin-tuck posture) has been recommended as a possible compensatory strategy to reduce aspiration of thin liquids by promoting airway protection during swallowing. However, a precise anatomical definition is lacking^11^ and evidence of effectiveness is equivocal being limited by variations in study designs and the reporting of conflicting results^12–14^. Another challenge is the requirement of the individuals with PD to be able to remember and follow their therapist’s precise instructions, which are not easy once they have to perform these manoeuvres unsupervised in their own environment^8^. However, one large, randomised study involving 711 people with PD indicated that using the chin-down posture eliminated the aspiration of thin liquids in approximately half the subjects, as shown by video fluoroscopic swallow studies (VFSS), and was the preferred technique by most subjects^15^. The flexion of the head in a chin-down posture enhances the closure of the laryngeal vestibule while swallowing, resulting in the protection of the airway by tilting the epiglottis downward, reducing horizontal movement of the hyoid bone, thus promoting the early laryngeal vestibule closure, which is particularly beneficial for individuals with delayed onset of laryngeal vestibule closure, as is common in people with PD^14,16^.

Employing the chin-down manoeuvre when drinking was shown in a systematic review and meta-analysis to reduce the risk of aspiration, pharyngeal residue, and to decrease the maximum swallowing pressure at upper oesophageal sphincter in individuals with dysphagia, including those with PD^13^. However, these compensatory strategies are not effective in everyone with PD and should be evaluated on a case-by-case basis^8^. The chin-down manoeuvre is likely to be difficult for individuals with PD for many reasons, including the effects of ageing, dementia in some individuals, neck rigidity, and abnormal neck and axial postures that could limit the abilities to perform this manoeuvre effectively. One of the challenges of applying the chin-down manoeuvre is that it requires conscious effort, so it is difficult for people with cognitive impairment or reduced self-awareness to undertake^17^.

In clinical trials, the efficacy of chin-down manoeuvre has been evaluated by different methods, varying from clinical rating scales, technology-based measurements, and ultimately VFSS, regarded as the gold standard tool that permits direct visualisation and objective evaluation of swallowing events^12,15,18–20^. However, VFSS is not routinely performed in clinical practice in people with dysphagia as it requires expertise to perform the procedure and to interpret the results^21^. To overcome the limitations in access to VFSS, recent developments have identified neck flexion angle, which can be verified by either the anatomical landmarks on radiographic images or by using a tracking algorithm that automatically extracts neck angles from sagittal videos of swallowing, as a representative marker of posture where aspiration of thin liquids can be prevented with chin-down manoeuvre^12,22^. The latter approach has been incorporated into a mobile application (NeuroPostureApp^©^) that can be used to evaluate postural deviation in PD with good reliability^23^.

While ‘safe drinking’ is a seemingly simple goal, there are several ‘real-life’ practical difficulties in its effective execution. It is unlikely that we can fix the underlying degenerative process of swallowing dysfunction in PD. Added to this, under supervision, while many people with PD may be able to perform chin-down manoeuvre every time they drink, they are less likely to do this consistently when unsupervised. It is therefore necessary to look for alternative solutions to address the problem and, rather than ‘fixing’ the person, find ways to improve their environment for example with assistive devices designed to alleviate reliance on consistent, correct postures and methods with each drink.

In developing an assistive device to promote safe drinking in PD patients with dysphagia, we have applied the fundamental design principle of ‘know thy user’^24^. User knowledge can be acquired by understanding what individuals with PD need, their preferences, abilities, motivations, and limitations, and then integrating these insights into the product design. Using the design philosophy of ‘form follows function’ we aimed to design an anti-choking mug that people with PD can drink from using a chin-down posture but without exerting any special effort^25,26^.

This manuscript describes the development process of the anti-choking mug which includes an in-depth analysis of the needs of the end user (the person with PD), incorporation of these finding into the design of the mug, and the testing of the final mug prototype. The primary objective of the study was to evaluate the effectiveness of anti-choking mug in people with PD in terms of reduced choking by objective measures (neck flexion angles) and the use of clinically validated scales.

## Methods

### Development of the anti-choking mug

#### Implementation of user-centred design

**Figure 1:**
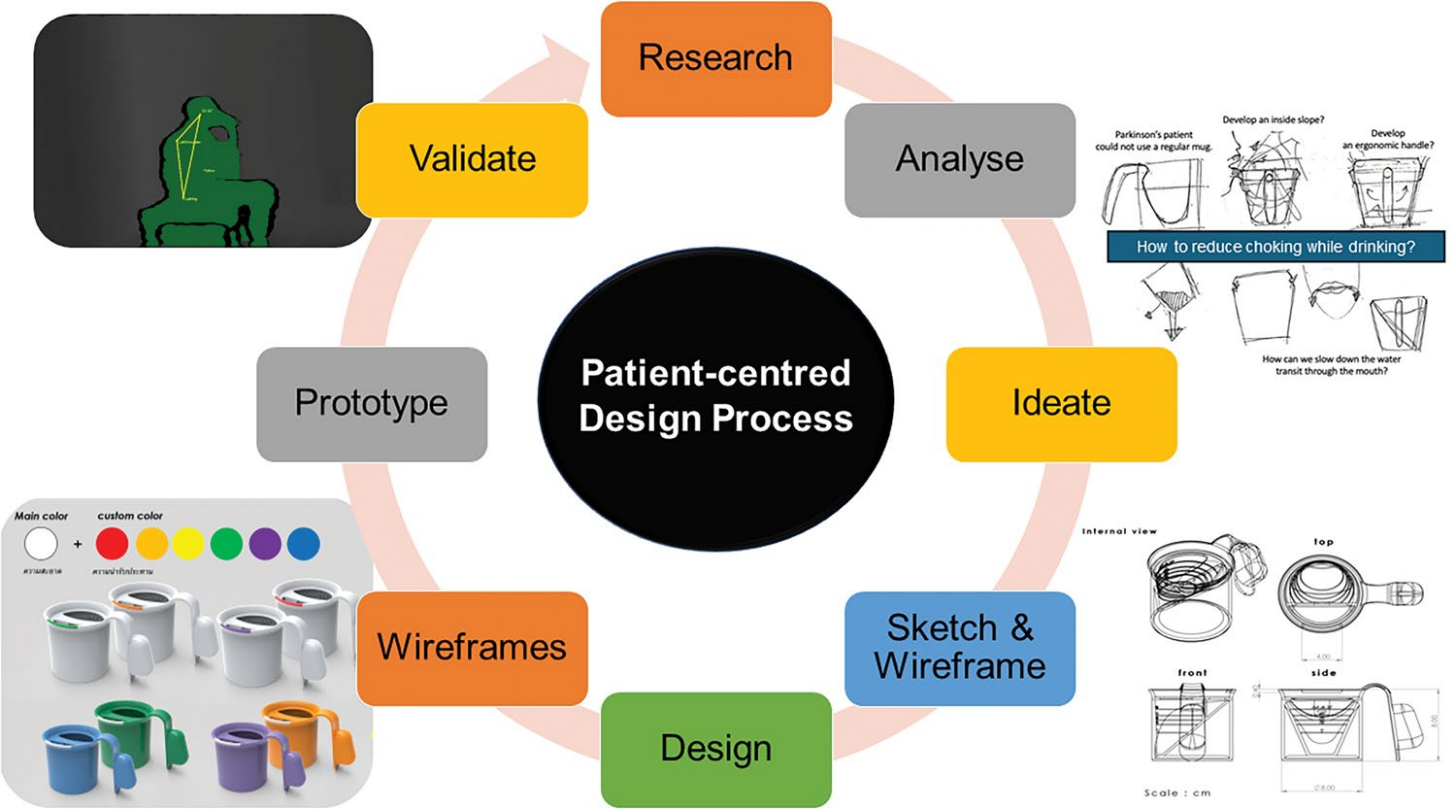
The cyclical user-centred, iterative design process implemented for the development of the anti-choking mug.

In the design and development process for the anti-choking mug, a cyclical, iterative user-centred design (UCD) approach was adopted involving input at each stage from people with PD who would be likely users of the final product in order to gain a deeper understanding of their needs, usual drinking tasks, and environments (Fig. 1)^27^. Designing devices for people with PD must be inclusive in order to accommodate a wide range of physical and cognitive abilities as well as focusing on simplicity, flexibility, and ease of use. In order to achieve a holistic approach, we brought together a multidisciplinary team with diverse expertise, including a movement disorder neurologist, a product designer, an engineer, and, most crucially, individuals with PD. Movement disorder neurologists offer invaluable medical insights into the intricacies of the disease, ensuring that the design addresses specific challenges related to oropharyngeal dysphagia and aspiration. Product designers contribute their creativity and expertise in crafting user-friendly and aesthetically pleasing solutions, while engineers bring technical proficiency to translate concepts into functional prototypes. However, the inclusion of people with PD in the development process is paramount. Their first-hand experiences as the ultimate users of the product provide an invaluable and unique perspective, guiding the team towards solutions that are effective and resonate with the practical needs and preferences of those living with PD.

We followed established product design processes^28^, beginning with defining the problem of aspiration of thin liquids, conducting research demonstrating limited evidence for proactive and consistent use of the chin-down manoeuvre, followed by ideation and conceptualisation of how we could encourage adoption of the chin-down manoeuvre within the design of a drinking cup that people with PD can use daily.

#### Anthropometric and disease-related considerations

We began the process by reviewing the literature on the drinking action of people with PD, of which, in the oral stage, there is the typical backwards-forward movement of the bolus, resulting in the rocking-rolling festination phenomenon that is related to the rigidity of the muscles responsible for lowering the back of the tongue to allow for the bolus or fluid passage to the pharynx^1,29^. During the pharyngeal phase, reduced tongue base retraction and pharyngeal wall constriction are frequently involved in pharyngeal residue, which may result in aspiration. Sip volume is another factor to consider, where the recommendation for cup sipping is about 25 ml per sip for males and 20 ml for females^30^. Impaired hand function that encompasses poor manual dexterity, reduced grip strength, and low self-perceived hand functional ability can result in delays in hand opening and pauses between the reach-grasp and take-to-lip parts of the drinking action^31–33^. Changes in hand grip anthropometry in PD is another consideration, as decreased hand grip strength is associated with decreased muscle mass and predicts functional limitations in the individuals^34^. Importantly, impaired neck posture during meals due to muscle rigidity has been identified as one of the important contributors to prepharyngeal dysphagia in PD patients^35,36^. Indeed, people with PD who have neck rigidity or abnormal neck postures may find chin-down manoeuvre a significant challenge to perform as part of their routine activities. An abnormal breathing-swallowing pattern in which people tend to perform inhalation instead of exhalation right before and after swallowing may also contribute to aspirations^37^. Last but not least, the effects of ageing should not be ignored as older adults reported that their hands' strength and dexterity have changed^38^.

#### Defining end-user needs

**Figure 2:**
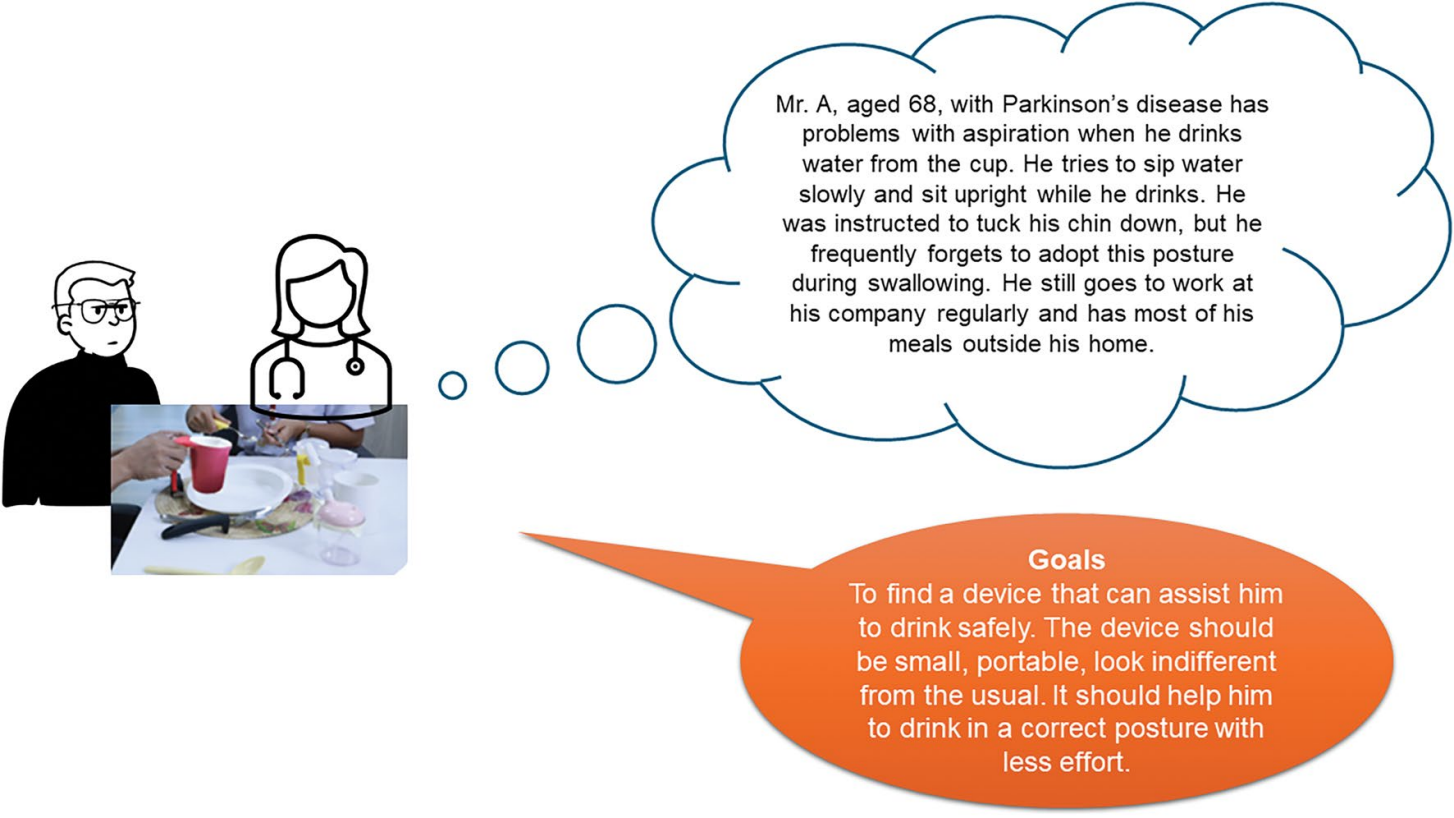
An example of the different ‘personas’ developed to represent potential target end-users that illustrate the practical problems that can result in the aspiration of thin fluids and the associated goal for device development.

Different ‘personas’ representing target end-users were developed to illustrate and define the problem of aspiration of thin fluids (Fig. 2). Subsequently, the ideal features of an anti-choking mug were ascertained from subject matter interviews with 30 people with PD and their carers. Thirty individuals with PD, recruited by a purposive sampling strategy from the outpatient clinics of the Chulalongkorn Centre of Excellence for Parkinson’s Disease and Related Disorders (ChulaPD; http://www.chulapd.org) were interviewed by treating neurologists to capture information about education, income levels, and living arrangements; all subjects had experienced symptoms of aspirations of thin fluids. The primary objective of these interviews was to comprehensively understand the unique challenges and requirements faced by this specific demographic in order to inform the design of anti-choking mugs. The interviews used semi-structured topic guides that focused on three main questions: (1) Techniques that they use to reduce the possibility of aspiration of thin fluids, (2) Devices that they have used to reduce the possibility of aspiration, and (3) Features that they would look for in a drinking device that would help reduce the possibility of aspiration.

Brief clinical demographics of the 30 subjects and a summary of their responses to the interview questions are summarised in Table 1. Most individuals with PD employed self-adjusted techniques to mitigate the risks associated with aspiration of thin fluids, with sipping water slowly as the most prevalent method (83.3%). Consciously pacing their intake of fluids underscores the importance of personalised coping mechanisms in reducing the risks of complications and enhancing their quality of life. Over 70% of individuals with PD reported using the chin-down manoeuvre to drink fluids to minimise the risk of aspiration. On the other hand, 26.7% of interviewees reported using straws to drink liquids, believing that it could lessen the risks of aspiration, while only 10% reported using nosey cups (cups with a special cutout to help maintain the proper head and neck positioning when swallowing) as their daily drinking appliances. The findings from preferences regarding features in their drinking devices to mitigate the risks of aspiration highlight several key aspects. Foremost among these is the preference for a slow and controllable water flow during drinking (56.7%). Following closely is the importance of the cup’s edge ergonomically fitting with the shape of the person’s mouth, emphasising the significance of comfort and ease of use in facilitating safe drinking experiences (43.3%). Lastly, portability emerged as another sought-after feature, underscoring the practicality of having a drinking device that can be conveniently carried and used in various settings.

**Table 1:** Clinical characteristics of users (individuals with Parkinson’s disease) who underwent interviews to help identify end-user needs for safe drinking. Values shown are mean ± standard deviation unless otherwise stated.

Parameter	Individuals with PD (n = 30)	Range
Age	71.4 ± 8.43	48–83
Sex		
Male	16 (51.6%)	
H & Y stage in the ‘ON’ state	2.97 ± 0.61	2–4
Disease duration	11.3 ± 3.05	7–20
Numbers of aspiration (times/month)	9.8 ± 6.47	2–24
Techniques subjects use to reduce aspiration, n (%)		
Sip water slowly	25 (83.3%)	
Tuck the chin down	22 (73.3%)	
Sit upright	20 (66.7%)	
Avoid talking while drinking	12 (40%)	
Devices they have used to reduce aspiration, n (%)		
Would like to have a new device that can reduce aspiration	12 (40%)	
Straw	8 (26.7%)	
Nosey cup	3 (10%)	
Plain cup	7 (23.3%)	
Characteristics of a device that could help reduce aspiration, n (%)		
Slow and controlled delivery of liquids	17 (56.7%)	
Adjustable edge to fit the person’s mouth shape	13 (43.3%)	
Portability	8 (26.7%)	

*H & Y* Hoehn & Yahr, *PD* Parkinson’s disease.

In addition to the primary features identified, individuals with PD provided feedback for additional features they would like to see incorporated into anti-choking mugs. One prominent request was for improved stability of the mugs, particularly to accommodate hand tremors. Enhancing stability enhances confidence in handling the mug and reduces the risk of spills, offering reassurance and promoting independent use. Given the importance of medication adherence in managing Parkinson’s symptoms effectively, incorporating a reminder system directly into the mug was considered to be a practical solution. Such a feature would help individuals stay on track with their medication schedules, ensuring timely intake and potentially minimising fluctuations in symptom management.

These insights obtained from the interviews highlight the multifaceted needs of individuals living with PD and underscore the importance of designing anti-choking mugs that address not only swallowing difficulties but also broader aspects of activities of daily living and disease management.

#### Prototyping and iterative design

Once user needs were identified and understood by the multidisciplinary team, the iterative process from concept generation to prototype development and simulation commenced. The anti-choking mug prototype was developed to specifically address the needs identified through the expert interviews with people with PD, supplemented with additional inputs from healthcare professionals in the multidisciplinary team. The anti-choking mug subsequently underwent further iterative modifications based on feedback gleaned from real-life experience of its use by individuals with PD and taking into account the ergonomic and anthropometric changes associated with the disease that may affect grip strength, dexterity and motor control.

Specific features of the final mug prototype were carefully designed to enhance the drinking experience, including a reduced rate of water flow to limit volume intake and help minimise choking. Studies of subjects with dysphagia have shown that a safe sip volume for liquids is less than 20 ml^39^, highlighting the importance of volume limitation when drinking. The inner cup slope was created to facilitate water flow to the person's lips and to allow them to drink without tilting their head backwards, which can be a problem if they have neck rigidity, an edge that aligns closely with the contours of the person's lips to make it easy to take water into the mouth without spilling, a thickened handle to facilitate firm hand grip and a wide bottom for stability, which is especially useful in individuals with hand tremors. In addition, a discrete pill dispenser was integrated into the handle, a hidden feature that serves to promote medication adherence, ensuring users have convenient and discrete access to their medications while on the go.

**Figure 3:**
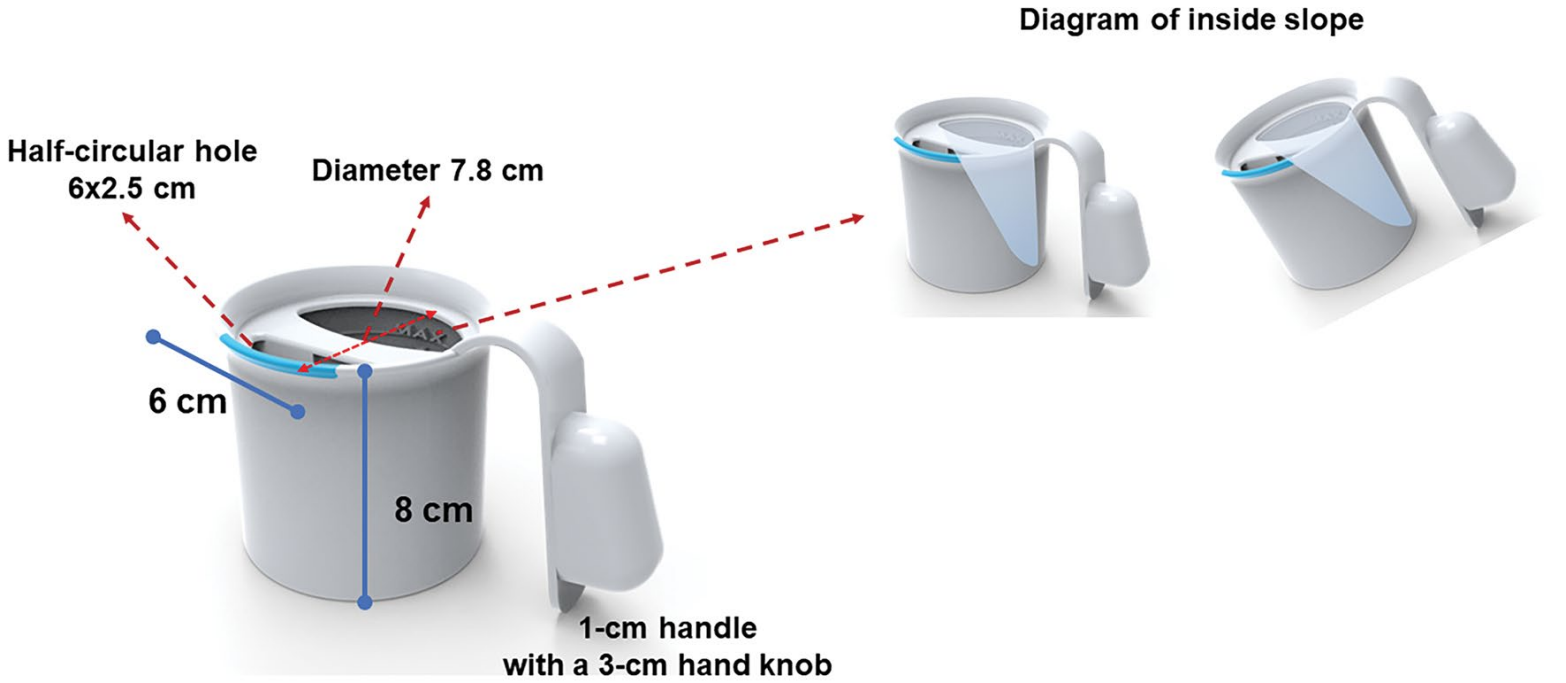
The design and specifications of the anti-choking mug.

The final design of the anti-choking mug is currently awaiting patent approval (patent pending) which limits the detail that can be provided here, but an overview is given below. The anti-choking mug prototype that was tested measured 7 × 10 × 12 cm (width × length × height) and was manufactured using a 3D printer (Original Prusa i3 MK3, Prusa Research a.s., Czech Republic) using polylactic acid, a plant-based material (Fig. 3). The cup's inner curved slopes, ranging from 17.5° (a necessary angle for an effective chin-tuck posture)^12^ to twice the neck flexion angle, were designed to enable smooth water flow to the individual's lips. This design allows for drinking without the need to tilt the head backwards, simulating the chin-tuck manoeuvre. A half-circular hole (2.5 × 6 cm) was introduced at the cup's edge to regulate water flow rate the sip volume, which does not exceed 20 ml. Considering anthropometric changes associated with hand grip in PD, the handle was 1 cm thick, featuring a 3-cm hand knob, designed to encourage firm hand grip. For the purpose of clinical testing, a sham mug was produced using printing methods and materials similar to those of the anti-choking mug. It was intentionally designed to be indistinguishable from the anti-choking mug when viewed from the outside, maintaining consistency in appearance. However, it lacked the innovative features found in the anti-choking mug, such as inner slopes and regulating holes.

### Testing of the anti-choking mug

#### Selection of participants for prototype testing

Between January 2023 and June 2023, 15 individuals diagnosed with PD according to the Movement Disorder Society (MDS) clinically established criteria were recruited from the Outpatient Clinic of the Chulalongkorn Centre of Excellence on Parkinson’s Disease and Related Disorders (ChulaPD, http://www.chulapd.org)^40^. To be eligible for inclusion, subjects needed to score a minimum of 3 (I choked at least once in the past week) on item 2.3 of Movement Disorder Society-Unified Parkinson’s Disease Rating Scale (MDS-UPDRS) part II, which evaluates swallowing difficulties (Over the past week, have you usually had problems swallowing pills or eating meals? Do you need your pills cut or crushed, or your meals to be made soft, chopped, or blended to avoid choking?)^41^. Individuals with unstable vital signs (heart rate ≤ 60 or > 100 beats/minute, systolic blood pressure < 90 mmHg or > 160 mmHg, or oxygen saturation < 95%) and those with a history of stroke, traumatic brain injury, amyotrophic lateral sclerosis, myasthenia gravis, or Lambert-Eaton syndrome, were excluded from the study. Additionally, individuals with abnormalities in the oral cavity contributing to dysphagia (such as cancer or a mass in the oral cavity, or a history of surgery in the oral cavity), history of severe aspiration (including cough or difficulty breathing after choking, difficulty breathing after eating, history of aspiration pneumonitis or bronchitis in the past year, or underlying respiratory disease such as asthma or chronic obstructive pulmonary disease), or cardiovascular disease (including coronary artery disease or arrhythmia) were also excluded. The Human Ethics Committee at Chulalongkorn University's Faculty of Medicine (Institutional Review Board No. 423/65) approved the research protocol, and written informed consent was obtained from all subjects prior to participating in the study.

#### Experimental protocol and study outcomes

Once participants had taken their usual PD medications and investigators had confirmed that they were in the ‘ON’ state, they were placed in a seated position one meter in front of a video camera (Sony Handycam^®^ CX405, Tokyo, Japan) that recorded at a rate of 60 frames per second. Subsequently, 20 ml of water was poured into both anti-choking and sham mugs, and participants were instructed to drink water randomly from each mug without prior knowledge of the mug types. This process was repeated twice for each mug by each participant. Neck posture alignment was assessed using photos from videos of a drinking task, depicting the neck posture in the sagittal plane. Two photos, each at the beginning and the end of individual drinking task, were selected, anonymised and blindly evaluated by two independent raters (RB and WP). The same raters reassessed photos of 10 randomly selected participants four weeks later to determine intra-subject test–retest reliability.

Safety precautions were considered essential throughout the intervention. Participants' oxygen saturation and heart rate were continuously monitored, and if any unstable vital signs occurred as per the exclusion criteria, the trial was immediately ceased. Additionally, all cups underwent sterilisation following standard health protocols, adhering to guidelines provided by the Thai Ministry of Public Health to prevent the spread of COVID-19.

**Figure 4:**
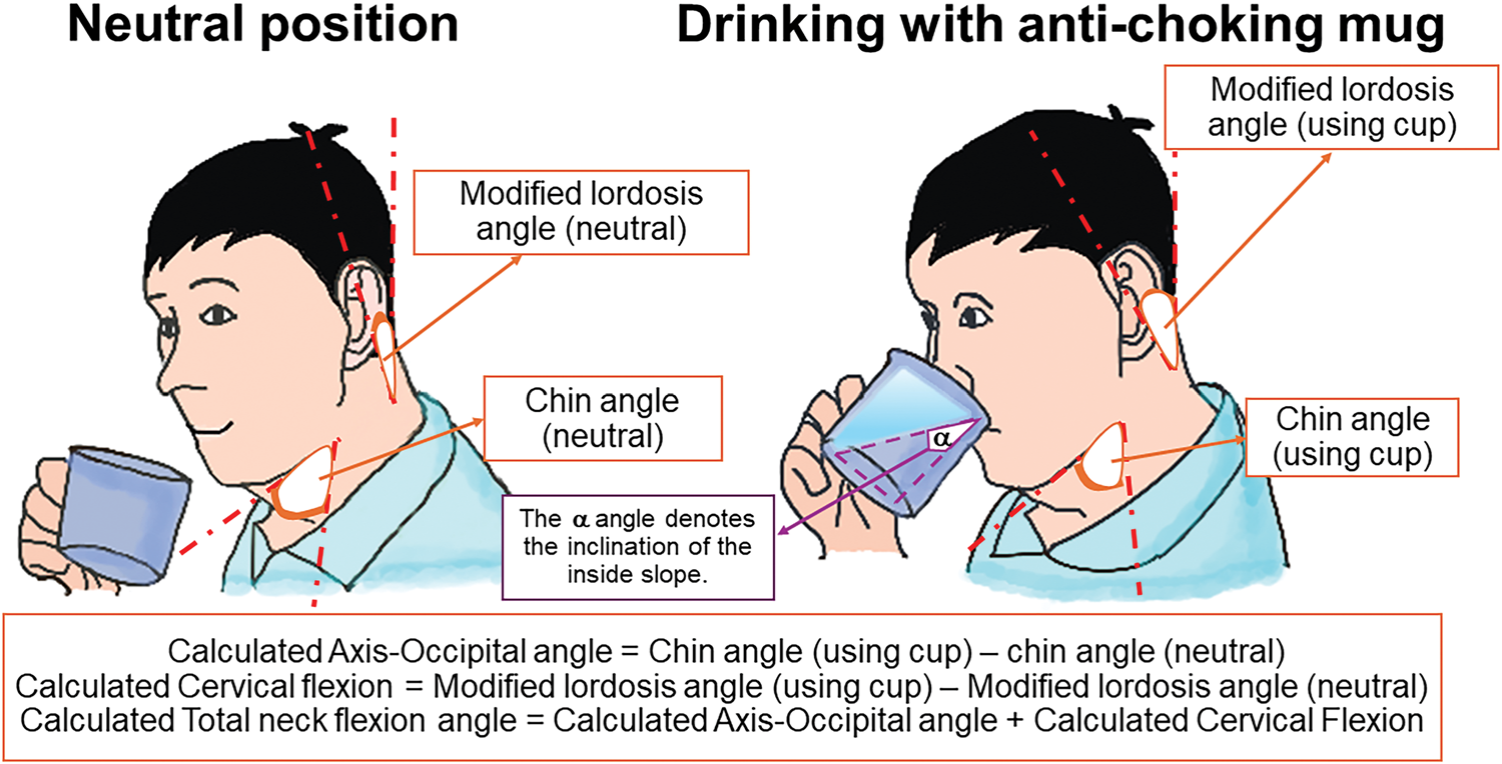
Comparison of neck flexion angles when drinking from a normal cup versus the anti-choking mug. The primary outcome measure of the study was the neck flexion angle, which comprises the axis-occipital angle and cervical flexion. The axis-occipital angle was calculated as the difference between the chin angle when using the anti-choking mug and the neutral position. The cervical flexion was determined as the difference between the modified lordosis angle when using the anti-choking mug and the neutral position. The α angle denotes the inclination of the inside slope of the mug.

In this study, neck flexion angle was utilised as the primary objective outcome to demonstrate the effectiveness of the anti-choking mug. This assumption was based on a prior study that used neck flexion angle to find an effective posture in which aspiration was prevented with the chin-down manoeuvre, demonstrating that 17.5° of neck flexion was a minimum required angle to achieve an effective chin-tuck posture^12^. With the use of the NeuroPostureApp^©^ (http://www.neuroimaging.uni.kiel.de/NeuroPostureApp), different neck angles could be identified^12,23^, based on previously published criteria and the application of surface anatomy corresponding to designated cervical vertebrae^42,43^. Definitions of chin angle, modified lordosis angle, axis-occipital (AO) angle, cervical flexion angle, and neck flexion angle are provided in Table 2 and illustrated in Fig. 4. The neck flexion angle, as the primary outcome, is determined by the sum of the AO flexion and cervical flexion angles.

**Table 2:** Definition of neck angles.

Angles	Definition	Surface anatomy
Chin angle	The angle between the lower margin of mandible and anterior surface of second cervical vertebral body	The second cervical vertebral body correlates with the surface anatomical landmark at the angle of the mandible
Modified lordosis angle	The angle between two lines drawn along the posterior margin of second (anatomical landmark at occiput^42,55^) and sixth cervical vertebrae (anatomical landmark at the cricoid cartilage^42,55^)	The posterior margin of the second cervical vertebra correlates with the surface anatomical landmark at the posterior margin of the occiput The sixth cervical vertebra correlates with the surface anatomical landmark at the cricoid cartilage
AO (axis-occipital) angle	The difference of chin angle between the neutral position and when the person uses the anti-choking mug	
Cervical flexion	The difference of modified lordosis angle between the neutral position and when the person uses the anti-choking mug	
Neck flexion angle	The sum of AO angle and cervical flexion

Secondary outcomes include both objective and clinical measures. Objective outcomes comprised AO and cervical flexion angles, providing additional insight into neck posture during water consumption as well as the total drinking time. Clinical outcomes include the SCAS-PD, consisting of 12 items that identify the occurrence of alterations in the oral and pharyngeal phases of swallowing. The SCAS-PD was recently validated in comparison to the VFSS, the gold standard method, demonstrating a high sensitivity (100%), specificity (87.5%), and good concordance (weight kappa concordance rate of 0.71), confirming its utility as a simple and cost-effective tool for evaluating dysphagia in PD^18,44^. In addition, cut-off points for SCAS-PD scores are available to define the severity of dysphagia: normal is a score of ≤ 2, functional swallowing is > 2 to ≤ 15, mild dysphagia is > 15 to ≤ 35, moderate dysphagia is > 35 to ≤ 60, and severe dysphagia is > 60^18^. As part of the item# 7 of the SCAS-PD (altered cervical auscultation), Littmann Classic II Paediatric Stethoscope was used to determine if there were sounds during the respiration, swallow, and respiration sequence that had not been observed before the drinking tasks.

#### Statistical analyses

The sample size for this study was determined based on a 95% confidence interval and a prevalence rate of 36.9% for dysphagia in people with PD. Considering a projected dropout rate of 10%, our calculations indicated that a total of 10 people with PD would be required for a feasibility pilot study. Baseline characteristics and the numbers of previous aspirations were analysed using means and standard deviations (SDs), or frequencies and percentages. Unpaired t-tests or Mann–Whitney *U* tests were used to compare demographic and continuous data between participants who used anti-choking mugs and sham mugs, depending on the normality of the distribution. The average of objective outcomes performed under two drinking tasks was used for statistical analysis. Chi-square tests or Fisher’s exact tests were applied to compare categorical data between groups. Exploratory correlations were conducted between age, MDS-UPDRS Parts II and III, total drinking time and the SCAS-PD and the total neck flexion angle using Spearman’s correlation. All statistical analyses were performed using SPSS software version 23 (IBM Corporation, New York, USA). *p*-values were defined as statistically significant at *p* < 0.05 in a two-tailed test.

### Ethics declaration

This study was conducted according to the Declaration of Helsinki, with the Human Ethics Committee at Chulalongkorn University’s Faculty of Medicine (Institutional Review Board No. 423/65) approval. Written informed consent was obtained from all subjects prior to participating in the study.

## Results

**Figure 5:**
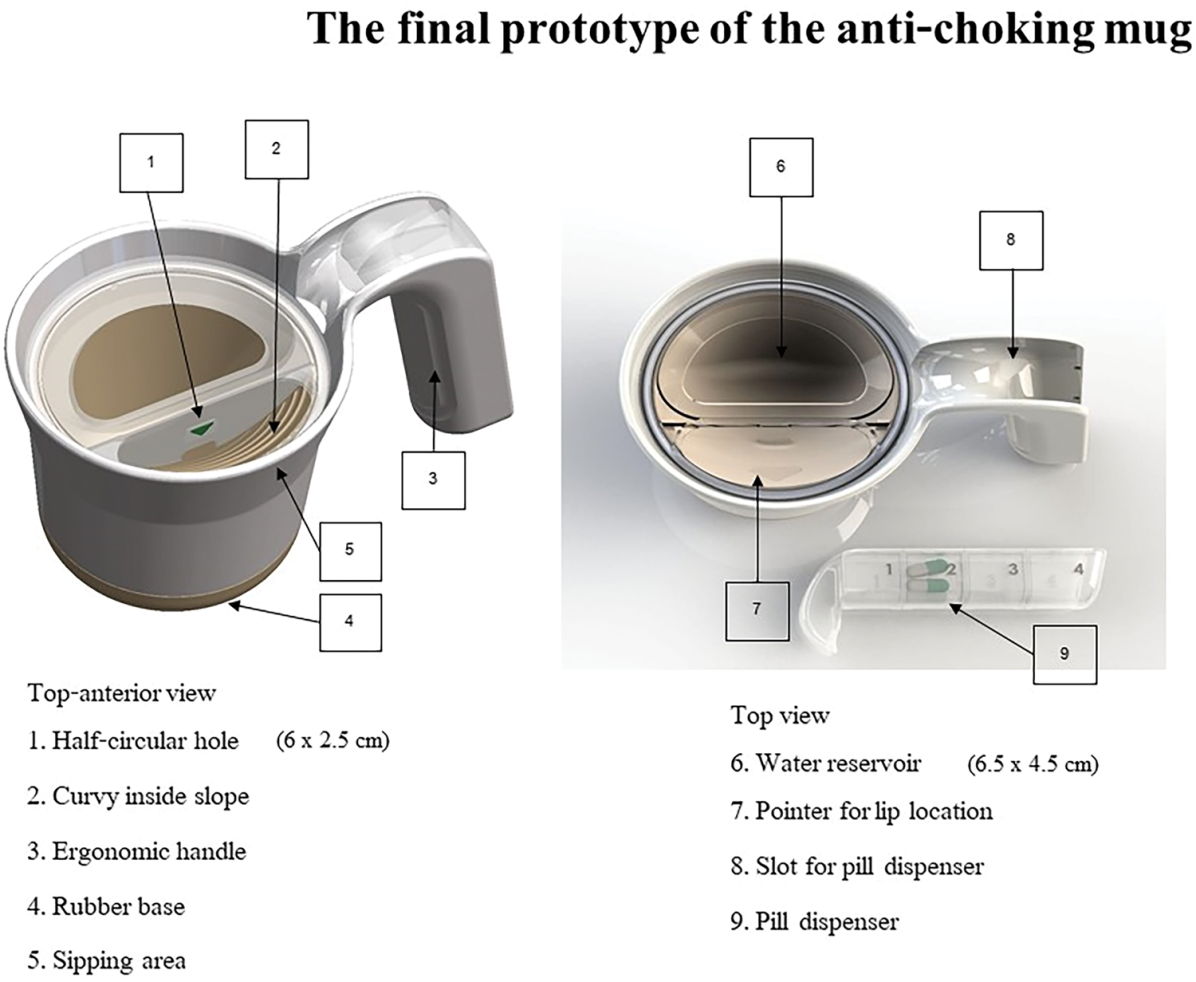
Final prototype of the anti-choking mug used for objective and clinical efficacy testing.

Fifteen individuals with PD (9 males, 6 females; mean (± SD) age 72.2 ± 8.83 years) participated is a study to test the final prototype of the anti-choking mug (Fig. 5). No subjects experienced any adverse events and there were no dropouts. Demographic data and disease characteristics of all subjects are shown in Table 3. The mean disease duration was 10.6 ± 3.79 years with a mean Hoehn & Yahr stage of 3.1 ± 0.8. The mean MDS-UPDRS Part II and III scores during the ‘ON’ period were 20.33 ± 11.78, and 32.93 ± 17.89, respectively. A comparison of the neck flexion angle and other objective and clinical outcomes of participants who performed drinking tasks with anti-choking mugs and sham mugs are shown in Table 4. We found that individuals with PD exhibited a significantly higher mean degree of neck flexion when drinking water from anti-choking mugs compared to sham mugs (26.87° ± 5.13° vs. 11.93° ± 8.88°; *p* < 0.001). Similar significant differences were also observed with AO (*p* = 0.001) and cervical flexion angles (*p* < 0.001). There were no significant differences of the chin and modified lordosis angles when comparing between the two mug types. Moreover, individuals with PD had a significantly longer drinking time when using anti-choking mugs in comparison to sham mugs. SCAS-PD was also significantly improved when subjects drank with the anti-choking mugs (Supplementary file 1). A mean score of 7 was achieved, which is within the range for ‘functional swallowing’ whereas a mean score of 49 was achieved when using sham mugs, indicating ‘moderate dysphagia’.

**Table 3:** Demographic data and disease characteristics of the 15 individuals with PD who participated in prototype testing. Values shown are mean ± standard deviation, except for gender.

Clinical parameter	Values
Sex, n (%)	
Male	9 (60%)
Female	6 (40%)
Age (years)	72.2 ± 8.83
Numbers of aspiration (times/month)	7.2 ± 6.56
Disease duration (years)	10.6 ± 3.79
Levodopa-equivalent daily dose (mg)	637.15 ± 316.56
H & Y stage in the ‘ON’ state	3.1 ± 0.8
MDS-UPDRS score	
Part II	20.33 ± 11.78
Part III	32.93 ± 17.89
TMSE scores	25.33 ± 2.47

*PD* Parkinson’s disease, *TMSE* Thai Mental State Examination, *H & Y* Hoehn & Yahr, *MDS-UPDRS* Thai-translated versions of the Movement Disorder Society-Unified Parkinson Disease Rating Scale.

**Table 4:** Comparison of primary and secondary outcomes between the anti-choking and sham mugs**.** Values shown are mean ± standard deviation.

Parameters	Individuals with PD (n = 15)		*p*-value
	Anti-choking mug	Sham mug	
Primary outcome			
Neck flexion angle (°)	26.87 ± 5.13	11.93 ± 8.88	< 0.001*
Secondary outcome			
Secondary objective parameters			
Total drinking time (seconds)	21.17 ± 13.13	12.58 ± 11.48	0.001*
Neutral position			
Chin angle (°)	65.23 ± 9.28	62.6 ± 12.06	0.967
Modified lordosis angle (°)	11.27 ± 2.25	11 ± 3.02	0.935
AO angle (°)	16.8 ± 3	7.47 ± 6.65	0.001*
Cervical flexion (°)	10.2 ± 3.5	4.47 ± 3	< 0.001*
Secondary clinical outcome			
SCAS-PD scores	7.08 ± 13.31	49 ± 27.73	< 0.001*

Comparison between anti-choking and sham mugs was performed using the Mann–Whitney *U* test *Significant findings (*p*-values < 0.05); *PD* Parkinson’s disease, *AO angle* axis-occipital angle, *SCAS-PD* Swallowing Clinical Assessment Score in Parkinson’s Disease.

Assessment of correlation was performed between the neck flexion angle and clinical parameters, including age, MDS-UPDRS Parts II and III, total drink time, and SCAS-PD scores (Table 5). A strong and significant positive correlation was demonstrated between the neck flexion angle and the total drinking time (r = 0.621, *p* < 0.001). A strong significant negative correlation was observed between the neck flexion angle and age (r = − 0.618, *p* = 0.014). Moderate significant negative correlations were also observed between total neck flexion angle and MDS-UPDRS Part II (r = − 0.555, *p* = 0.032) and SCAS-PD (r = − 0.559, *p* = 0.001), respectively.

**Table 5:** Correlations between neck flexion angles, SCAS-PD and clinical parameters.

Parameter	Age		MDS-UPDRS part II in the ‘ON’ state		MDS-UPDRS part III in the ‘ON’ state		Total drinking time (seconds)		SCAS-PD score	
	r_s_	*p*-value	r_s_	*p*-value	r_s_	*p*-value	r_s_	*p*-value	r_s_	*p*-value
Neck flexion angle	− 0.618*	0.014	− 0.555*	0.032	− 0.044	0.876	0.621*	< 0.001	− 0.559*	0.001
SCAS-PD score	− 0.005	0.985	0.566*	0.028	0.535*	0.04	− 0.338	− 0.338	–	–

All correlation analyses were performed by Spearman’s correlation (r_s_); *Significant findings (*p*-values < 0.05); *PD* Parkinson’s disease, *MDS-UPDRS* Thai-translated versions of the Movement Disorder Society-Unified Parkinson Disease Rating Scale, *SCAS-PD* Swallowing Clinical Assessment Score in Parkinson’s Disease.

## Discussion

It is recognised that in older people, human factors and ergonomics become increasingly important in undertaking daily tasks due to the progressive limitations of this population, and this needs to be reflected in the design of products they use^45,46^. We therefore employed a UCD approach that focused on the specific needs of individuals with PD in our product development process and study design. Using this UCD approach, we were able to successfully develop an anti-choking mug that integrates user needs and limitations into its design. Our tested prototype was able to effectively reduce aspirations, as determined by a validated clinical scale, the SCAS-PD. The mechanisms by which the aspirations were reduced were likely to be several, first related to a more effective posture of the individuals as shown by a significantly larger neck flexion angle in those using anti-choking mugs compared to those with sham mugs. As the evidence suggests that oral transit time is shortened with chin-down manoeuvre^13^, a significantly longer drinking time demonstrated in our study with anti-choking mugs is likely to reflect an effective control of water flow within the mug due to the curved surface of the inside slope and the half-circular hole, located at the cup's edge that regulates the water flow rate and the sip volume. The revised version of the prototype also includes other supportive mechanisms include increased stability of individuals with PD when holding anti-choking mugs as a result of ergonomically designed handles that incorporate anthropometric changes of hand grip in PD and a hidden pill dispenser (Fig. 5). While anthropometric changes have been partly studied in older adults^38^, we are unaware of any studies in PD that have explored these human-body dimensions in terms of movement control previously^34^.

Overall, the combined design features of the final prototype of the anti-choking mug allow people to drink fluid with a significantly increased neck flexion angle, which is shown in our study to be associated with an improved Swallowing Clinical Assessment Score in Parkinson’s Disease (SCAS-PD) score. This posture is likely to mimic the chin-down manoeuvre so subjects are less likely to be in a neck extension posture, which studies have suggested is the most difficult position for effective swallowing^47^. During neck extension, there is a mechanical widening of laryngeal vestibule and narrowing of valleculae, leading to a decrease in upper oesophageal sphincter relaxation making it difficult to achieve closure^47,48^. In contrast, during the chin-down manoeuvre, due to flexion of the neck and the resulting head position, decreased distances are observed between anatomical structures in the pharynx, which may contribute to the effectiveness of this technique as it shortens the route for laryngeal elevation and airway closure^49^.

We have also demonstrated in our study that increased neck flexion angle can be achieved when using the anti-choking mug as not only does it promote the chin-down manoeuvre, but also the patented design of the inner slope that allows water to reach the oral cavity sooner, providing an additional advantage for those with neck and upper trunk rigidity, as frequently encountered in individuals with PD. The mean neck flexion angle achieved with anti-choking mugs was 26.87°, beyond the minimum requirement of 17.5° of neck flexion to achieve an effective chin-down manoeuvre as demonstrated in a prior study^12^.

One important aspect of this study is that it incorporates new technology-based outcome measures to supplement standard clinical observations. Technology-based assessment is an emerging tool in PD clinical trials but currently, however, as yet few studies use this dual approach. Importantly, measurement of neck flexion angles with digital technology provides the options for studies of oropharyngeal dysphagia to be undertaken in the individual’s own home, rather than requiring them to attend the hospitals. Previous studies of swallowing efficiency and dysphagia in PD have generally used VFSS which is technically difficult, hospital based, and requires specialists to interpret the results. Clinical testing that implements technology-based outcomes to supplement information from standard clinical rating scales, like MDS-UPDRS, is something that future clinical studies in PD should consider when they evaluate interventions to improve the functional daily activities of people with PD as the primary outcome measure. Such studies should ideally be undertaken in the individual’s own environment to reflect real-world experience. Notably, in people with PD, functional ability to perform daily tasks is known to be closely associated with quality of life^50^.

A major strength of the study was that it placed the end-users of the product—individuals with PD—at the centre in the design of anti-choking mugs at all stages. Our multidisciplinary UCD approach ensured that the mug was developed and prototyped to address the specific needs of users and could effectively minimise aspiration. Related to this, dehydration is a common and under-recognised issue in the elderly, including people with PD. Part of this problem may be related to a fear of choking and also frequent toileting in these individuals. Therefore, it is hoped that an added benefit of the anti-choking mug will be to facilitate greater fluid intake in people with PD to improve both hydration levels and also orthostatic hypotension which is common in PD^51–53^.

Another important point when evaluating postural strategies for dysphagia relates to the precise definition of the chin-down, or chin-tuck, manoeuvre for which there is no consensus in terms of terminology or exact procedure^54^. A survey of speech and language pathologists in Japan and the United States found poor agreement about the meaning of the chin-down and chin-tuck manoeuvres^11^. Variations in the performance of the technique across studies makes it difficult to evaluate comparative efficacy. A strength of our study is that we were able to bypass this procedure by using the anti-choking mug itself to promote neck flexion when swallowing fluids. This approach may also help individuals with PD who have antecollis and find it difficult to drink fluids with a chin-down posture^8^.

This study was the first to evaluate the anti-choking mug, and so was undertaken in the hospital setting, rather than in the subject’s home, as safety was our priority and we wanted to ensure that no subjects experienced significant choking events. Another safety precaution put in place was strict hygiene to prevent the spread of COVID-19 amongst subjects, so rigorous sterilisation of test mugs was performed according to recognized standards. Patients were monitored throughout each test by the principal investigators, RB and WP.

Our study does have some inherent limitations. We did not use VFSS to assess swallowing, which is considered as a gold standard tool to evaluate dysphagia. However, we used neck flexion angles which have been studied in comparison with VFSS and found to be a valid marker to determine the swallowing efficiency^12^. As this was a feasibility study, prototype testing only included a small number of subjects, however future studies should consider testing in the individual’s usual place of residence, since the hospital setting does not reflect their real-life environment. In the prototype study, we only assessed the immediate effects of drinking using the anti-choking mug, and not its use over the longer-term. Usability testing should be included in future studies of the anti-choking mug to determine user feedback.

In summary, when designing an anti-choking mug, or any other assistive device, for people with PD, it is imperative to recognise and acknowledge the inherent challenges these individuals face, particularly concerning dysphagia and medication management. By embracing a paradigm that prioritises the experiences and needs of those directly affected by PD, we can better address the multifaceted nature of these challenges. Blaming individuals for dysphagia-related incidents or medication non-adherence overlooks the broader systemic factors at play, such as motor impairments, cognitive fluctuations, and sensory deficits, which significantly contribute to these issues. Therefore, instead of attributing errors solely to individuals, we felt it was essential to critically examine the design of the anti-choking mug, ensuring that it was not only compatible with the physiological and cognitive abilities of users but also accommodated the complexities of their daily lives. By shifting the focus from the requirements of the system and machinery to the requirements of people living with PD, we were able to develop an anti-choking mug that was truly effective in meeting the needs of the intended user, as well as fostering safety, independence, and enhancing quality of life.

### Supplementary Information

Supplementary Information.

## Data Availability

The datasets generated during and/or analysed during the current study are available from the Roongroj Bhidayasiri as a corresponding author on reasonable request.
