# Supplementary material for: User-centred design, validation and clinical testing of an anti-choking mug for people with Parkinson’s disease
Source: Sci Rep. 2024 Jun 19;14:14165. doi: 10.1038/s41598-024-65071-8 (PMC11187143; doi:10.1038/s41598-024-65071-8)
Supplement: Supplementary file 1 — Supplementary Information. [file 41598_2024_65071_MOESM1_ESM.pdf]

Score attributed to each event suggesting an alteration in the dynamic of swallowing in SCAS-PD (Loureiro, 2013)

Signs suggesting an alteration in swallowing found during assessment	Score	
	For each offering	Max. possible
ORAL PHASE		
Altered lip closure	1	3
Labial discharge	1	3
Prolonged oral transit time	2	6
Residue	2	6
PHARYNGEAL PHASE		
Multiple deglutition	2	6
Reduced larynx elevation	10	30
Altered cervical auscultation	10	30
SIGNS OF PENETRATION/ASPIRATION		
Throat clearing	10	30
Cough	15	45
Change voice quality	15	45
Choking	20	60
Alteration in breathing	30	90
Total		354
